# Supplementary material for: Hexapeptides from mammalian inhibitory hormone hunt activate and inactivate nematode reproduction
Source: PLoS One. 2022 Dec 1;17(12):e0278049. doi: 10.1371/journal.pone.0278049 (PMC9714824; doi:10.1371/journal.pone.0278049)
Supplement: S1 File — Figshare: Edman sequencing of biologically active fractions of sheep ovarian follicular fluid and blood plasma. https://doi.org/10.6084/m9.figshare.16432833. This project contains amino acid sequencing data and details how EPL001 and Candidate 7500 were found. (DOCX) [file pone.0278049.s001.docx]

**Supplementary Information 1 (S1)**

**Sequencing & Purification**

S1 is provided in support of ‘Hexapeptides from mammalian inhibitory hormone hunt activate and inactivate nematode reproduction’

| SEQ ID NO | DESCRIPTION | EDMAN SEQUENCES AND RESIDUE NUMBER | PROVENANCE AND OPERATOR COMMENTS |
| --- | --- | --- | --- |
|  |  | **1 2 3 4 5 6 7 8 9 10 11 12 13 14 15 16 17 18 19 20** |  |
| 1 | First Sighting | M M x V? x P V G? G x F L | Ovarian follicular fluid; spin filters, gel filtration, anionex, SDS-PAGE; ‘Possible sequence large numbers of interesting peaks’ |
| 2 | Second Sighting | M L/K P L T G Q A M E F | Blood plasma; ultrafiltration, 3-30 kDa ‘upstream precipitate’, SDS-PAGE |
| 3 | EPL001 | M K P L T G K V K E F N N I | Blood plasma; upstream precipitate; SDS-PAGE; ‘Relatively clean sequence’ |
| 4 | Beale 4 | x x P x x x x V/L x x F/K N x x | Reference plasma; spin filters, gel filtration, anionex, SDS-PAGE |
| 5 | EPL001 Extension | M K P L T/G K V K x F N N I K/I G F/ Y/D x F/V I/V I | Upstream precipitate, aqueous extract; SDS-PAGE; “Co-elution T/G artefact for TG; ‘shadowing latterly’, KGFxVI inferred.” |
| 6 | Harwell 1 | M K V/I T/G Q Y S/V G? K? | Upstream precipitate, tricine gradient gel |
| 7 | Harwell 2 | M N/F P/I/M L N V/A I T/P | Ditto |

**S1 Table 1.** **Ovine amino acid sequences in order of acquisition by automated Edman degradation.** Samples analysed at The Babraham Institute, Cambridge, UK (hereafter Babraham) except for two analysed at Harwell Laboratory, South Oxfordshire, UK (hereafter Harwell). SEQ ID NO = Sequence Identification Number. Anionex = anion exchange chromatography.


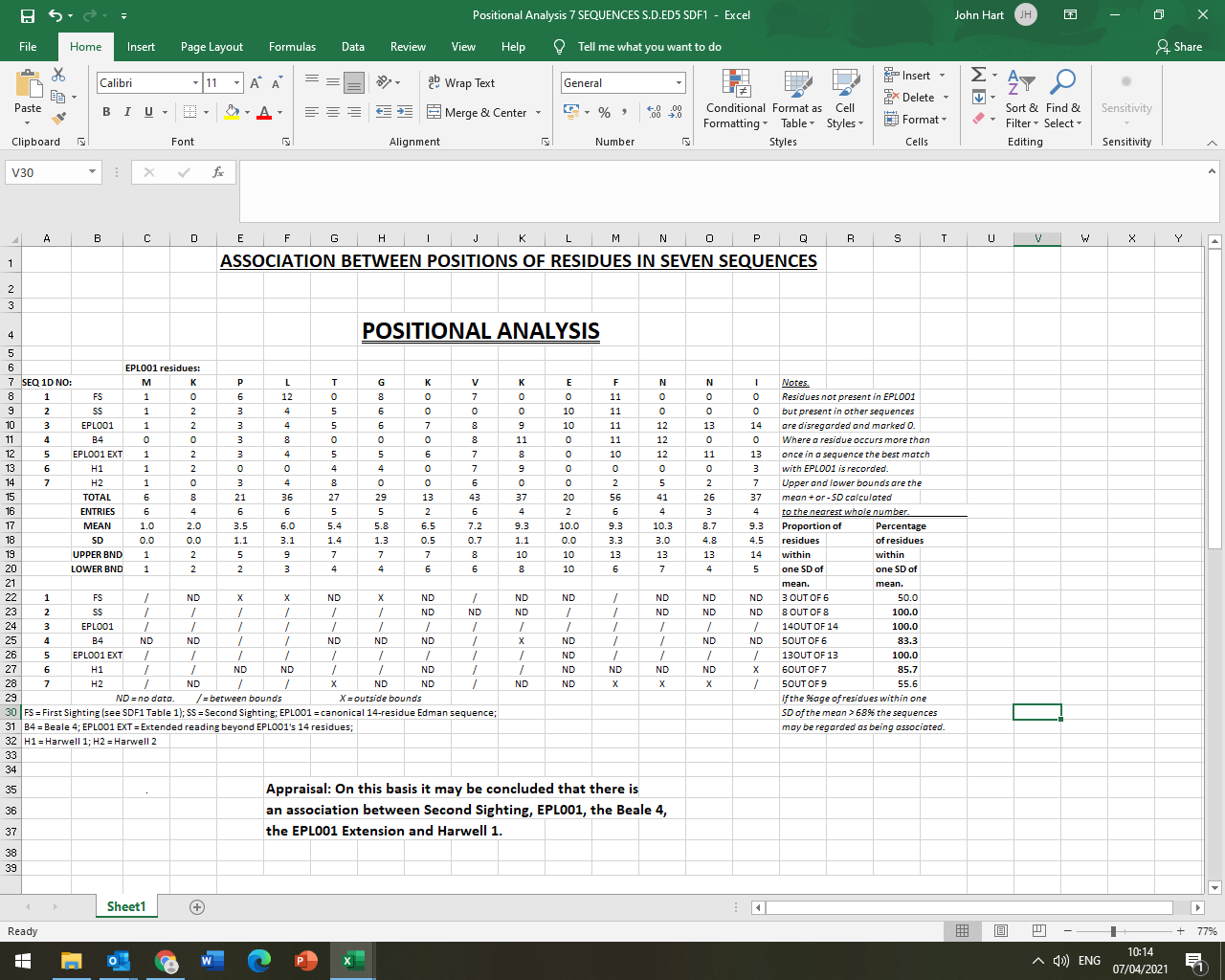


**S1 Table 2.** **Amino acid positional analysis.** The Edman-derived EPL001 N-terminal amino acid sequence MKPLTGKVKEFNNI was seen in whole (SEQ ID NO: 3, S1 Table 1; also Hart, 2013) or in part seven times in separate purification runs involving sheep material that was biologically active (i.e. anti-organotrophic in vivo and anti-proliferative in vitro) and contained gel and MS Candidate 7500, before Candidate 7500 was lost to view. Notable landmarks in S1 Table 1 sequences are an initial methionine, a position-3 proline, a mid-sequence valine and a position-11 phenylalanine. Five of the sequences (SEQ ID NOs: 2-6) have enough in common to support a mathematically validated association (S1 Table 2). There are 11 different aa in the 14-residue EPL001, given 3Ks & 2Ns. The two mathematical outliers comprise SEQ ID NO: 1, having 6 of the 11 EPL001 residues, in all its peculiarly rich sketchiness (see below), and SEQ ID NO: 7 in its chaotic near-completeness: 9/11. The impression from these two sequences, both of which lack EPL001’s charged Ks and an E, and from the other sequences in S1 Table 1, is of automated Edman sequencing struggling to gain traction.

[A description follows of the inhibitory hormone purification campaign.]

The prototype purification method, which proceeded on the assumption that a secreted item was sought, involved molecular weight sizing via spin and gel filtration, followed by anion exchange chromatography (Hart, 2003). Ovine fluids were obtained surgically; materials from other species were abattoir derived. Specifically, 120ml of ovine ovarian venous plasma was spun through a Centriprep-30 cartridge, which contains a size-selective permeable membrane. The nominal 0-30 kDa filtrate was then further spun through a Centriprep-10 cartridge to obtain a nominal 10-30 kDa fraction which was gel filtered using a Pharmacia Superdex-75 column to obtain a nominal 10-20 kDa fraction. This was applied to a Pharmacia FPLC Mono-Q anion exchange column, with a gradient of 0-0.3 M NaCl. Bioassay guidance was provided via organ mass reductions in female rats (e.g. affecting pituitary, ovaries, uterus, kidneys etc.) (Hart, 2003) and by inhibition of the proliferation and viability of rat bone marrow mesenchymal stem cells in culture, for example using Alamar Blue (Hart, 2001). Inhibitory activity in vivo (i.e. organ shrinkage) was demonstrated for an anion exchange fraction of ovarian venous plasma eluting at 0.1-0.2M NaCl, compared with jugular vein fractions from ovariectomised sheep (Hart, 2003). Similar effects were reproduced in two other laboratories. Analysis focused on anion exchange fractions of ovarian follicular fluid, abattoir derived, from sheep (S1 Table 1, SEQ ID NO: 1), pigs, goats and cows. To determine availability of material, anion exchange fractions of porcine ovarian follicular fluid of 0.5 ml containing Candidate 7500 material at *m/z* 7-8000 were concentrated using micro reversed-phase technology (Zip Tips, Millipore, Watford, UK, and Sep-Pak cartridges, Waters, Elstree, UK, used sequentially) to a volume of ~100 µl. Total protein content in the final sample was estimated at 1 µg, using a dye-binding micro-method (Bio-Rad, Watford, UK) (personal communication, Charles Dickerson, Harwell). Classically, peptides and proteins are provided for MS either in dissolved form for ESI (electrospray ionization) or co-crystalized with a matrix chemical in MALDI. No peaks were seen using ESI. More productive was MALDI-TOF (Babraham), which yielded ions in the range *m/z* 7-8000, dubbed Candidate 7500. These appeared in fractions shown to be active in the in vitro assay, with no optical density registered by the chromatography column UV detector and without band visualization in gel electrophoresis. Scale up involved jugular vein plasma from sheep, with activity validation again provided by the in vitro assay and dose-dependency demonstrated (Hart, 2001, Fig. 4 therein). The anion exchange elution profile was widened to 0-1.0 M NaCl, with the in vitro assay picking up activity in late eluting fractions (Hart, 2001) more reliably than in early ones, but with Candidate 7500 seen by MS in both. Fraction 29 of anion exchange separated plasma showed peaks at ~7800 in MALDI-TOF. This fraction was concentrated (Centricon-3) and run on reducing SDS-PAGE for analysis. No bands were evident using Coomassie Blue. The material was dried down, run again and blotted. The region indicated by MW markers to be around 7 kDa was cut out and sequenced using step-wise N-terminal Edman degradation (Edman, 1950; Smith, 2001) in automated form (Applied Biosystems Procise, Babraham). This gave a weak result, xxPxxxxV/LxxF/KNxx (S1 Table 1, SEQ ID NO: 4), which however turned out to be pivotal, involving as it did maximally purified material, making sense of other results.

A simplified purification procedure, for scale up and to reduce losses, involved ultrafiltration of sheep jugular vein plasma (up to 1.5 L, EDTA anticoagulated, follicular phase of oestrous cycle: Hart, 2001) through a 30 kDa Vivaflow tangential flow membrane. The filtrate was concentrated over a 3 kDa Millipore stirred cell ultrafiltration membrane, with the retentate (~25 ml of 3-30 kDa concentrate) separated using a Mono-Q 10/10 anion exchange column in HPLC. Candidate 7500 was identified in fractions active in vitro but in insufficient quantities for sequence determination. Serendipity asserted itself, with the appearance of a precipitate on the 3 kDa filter (Hart, 2013). This, like the supernatant and anion exchange fractions thereof, proved to be inhibitory in vitro. The ‘upstream precipitate’ was submitted to MALDI-TOF MS (Harwell). Bizarrely, all that flew from this variably discoloured multi-milligram apparent mixture was Candidate 7500. (The same was true when the upstream precipitate from bovine ovarian follicular fluid was subject to MS.) A sample of the upstream precipitate was separated by SDS-PAGE on a gradient gel under reducing conditions giving a large number of bands of which the lowest defined was ~7 kDa . This was designated Band 1 and yielded MALDI-TOF peaks of 7.5-8 kDa (personal communication, Pat Barker, Babraham). Trypsinisation for possible database identification resulted in a set of peptides but no identification. Edman sequencing of Band 1 gave a 14-residue N-terminal sequence deemed ‘relatively clean’: MKPLTGKVKEFNNI (S1 Table 1, SEQ ID NO: 3, EPL001). The match with the sequence obtained from the anion exchange fraction of plasma is xxPxxxxVxxFNxx, privileging V & F at positions 8 & 11, respectively. The residues **P∙V∙FN** were designated the ‘Beale 4’ (S1 Table 1, SEQ ID NO: 4), after the scientist responsible for purification (Dennis Beale, Babraham). Among the 557,397 proteins in the SwissProt division of the UniProt database, which is non-redundant and covers all taxons, there are 872 matches to xxPxxxxVxxFNxx (0.16% prevalence, 1 in 639), none at the N terminus (personal communication, Chris Mundy, co-author). This means that the probability of getting a Beale 4-containing protein by chance in successive purifications is 1 in 639^2^ = 1 in 408,321. The UniProt database for *Ovis aries* is also non-redundant and contains 23,110 proteins, but only 457 of these are present in SwissProt. This database supplies 65 matches to the Beale 4 14mer, none N-terminal (0.28% prevalence, 1 in 355, meaning the dual purification probability is 1 in 355^2^ = 1 in 126,025). Of these 65 none correspond to the dozen candidate proteins identified from an immunoprecipitation campaign involving rat hypothalamic extract exposed to an anti-EPL001 antiserum (Hart et al, 2017, Table 2 therein). None has a sequence resembling that of EPL001, beyond possessing the Beale 4 in register, and none has EPL001’s initial residue, methionine, two places before the Beale 4 proline. (Only 10 of the 65 candidates have methionine anywhere in their 14mer sequence.) Conclusion: the N-terminal Beale 4 residues are inscrutable without reference to the N-terminal EPL001, which is itself bioinformatically obscure.

Within the reverse spliced model of sSgII-70 formation (see the paper’s Discussion) the Beale 4, PVFN, includes the 9+61 junction, V-F, as in _1_MLKTGEKP**VF**K_11_. The Beale 4 residues tie the three sSgII shuffled-sequence modules together and are on the righthand side of the paper’s sequence grid zigzag, presumably betokening spatial proximity. There is an alternation in sSgII-11 between residues that are hydrophobic (shaded, with proline and glycine included here in this category) and lysines (underlined), with the Beale 4’s PVF representing a hydrophobic cluster (bold): MLKTGEK**PVF**K. Within the Edman Nonsequentialism concept (S8) P, V & F are picked from sSgII-70 in sequential order by the Edman machine but at cyclic intervals to yield their placement in EPL001: xx**P**xxxx**V**xx**FN**xx. A mass spectrum from the Beale 4 sample yielded a tussock of peaklets in the range c7500-c7900 Da. The question arises: Why this would yield a unitary Edman sequence at all, even a weak partial one? The response is that the peaklets are artefacts of MALDI-TOF MS. There is just sSgII-70, which sheds variable numbers of C-terminal residues in mass spectrometry, as will be reported elsewhere. The unification of the Beale 4 peaklets in this way connects EPL001 with sSgII-70. Meanwhile an explanation based on Edman Nonsequentialism is given elsewhere (S8) of the mixed signals V/L and F/K in the Beale 4 sequence and also of the mixed L/K signal in position 2 of the Second Sighting (S1 Table 1, SEQ ID NO: 2).

The First Sighting (SEQ ID NO: 1, S1 Table 1) arguably depicts an Edman machine giving a disordered rendition of EPL001, with hints of sSgII-70. There are apparent stutters, which look like machine artefacts: MM, V?xxV, G?G. There is an M-V-F correspondence with EPL001, the M & F in register, the V close, with no residues present in the First Sighting that are not found in EPL001. In First Sighting there is _1_**M**xxxx**PV**_7_, while in the second sorting domain of sSgII there is _367_**M**xxxxxx**PV**_375_ and in the fruit fly protein’s homologue of SgII’s second sorting domain there is _1048_**M**xxxxxxxxxxx**PV**_1061_). Referring to the First Sighting, the chance of selecting at random a 14-residue sequence containing M-PV is 1 in 1007 (S2). Based on a standard deviation analysis the mathematical association between SEQ ID NO: 1 and EPL001 falls below the acceptance threshold (S1 Table 2), yet the First Sighting nonetheless has in the form _6_**PV**xxx**F**_11_ the PVF of the Beale 4’s _3_**P**xxxx**V**xx**F**N_12_. There are three non-reads (x), in positions 3, 5 & 10. These call to mind the trio of lysines in sSgII-14 (positions 3, 7 & 11) and those in EPL001 (positions 2, 7 & 9), and hint at residues unreadable through involvement in proposed crosslinking. The Second Sighting of S1 Table 1 (SEQ ID NO: 2) is EPL001 half seen, while the EPL001 Extension takes the reading beyond the canonical 14 residues out to 20, adding a possible KGFxVI when artefactual ‘shadowing’ is allowed for (S1 Table 1, SEQ ID NO: 5, operator comment). The ‘continuation 20mer’ synthetic peptide EPL120 (i.e. the EPL001 14mer with KGFGVI added C-terminally) was obtained using aqueous extract of the upstream precipitate. The attempt to eliminate potential bacterial contamination reduced the precipitate but did not abolish it. Band 1 from an aqueous extract of potentially contaminated precipitate gave a MALDI-TOF major peak of *m/z* 7572, while that from a separate batch of ostensibly uncontaminated material gave a main peak at 7579.

Further purification runs were effected at a separate laboratory (Harwell), with Edman sequencing by a different machine (Applied Biosystems Procise 491, University of Leicester, Leicester, UK). This effort provided echoes of the EPL001 sequence in the form of MKxT/GxxVxK (SEQ ID NO: 6) and MxPLxV (SEQ ID NO: 7). The former is deemed mathematically associated with EPL001, the latter not (S1 Table 2). Yet if NNI is dropped from the EPL001 analysis, as relating to residues deemed non-contiguous, then SEQ ID NO: 7 approaches to within a couple of percentage points of the >68% acceptance threshold, as indeed does the First Sighting, SEQ ID NO: 1.

Purification continued with a variety of starting materials, deploying different antisera in various ways. Ovine median eminences were subjected to acid extraction, the results with 1M HCl being deemed best. The crude extract was then run down an RP-HPLC column, with dot blotting of fractions using a guinea pig anti-EPL001 antiserum. Immunoreactivity was confined to a single fraction. In a revised approach, acid-extracted mediance eminence material was first subjected to anion exchange chromatography, as in the canonical purification method, then RP-HPLC. Immunoreactivity was again confined to a single fraction (F19 of 24), as determined with the guinea pig antiserum and also with a goat (female) anti-EPL001 antiserum of superior sensitivity (yielding shorter development times in dot blotting). No Edman or MS/MS sequencing data were forthcoming from this exercise. Another purification procedure used an immunoaffinity column with an anti-EPL001 rabbit antibody linked covalently to the solid phase. Feedstock was 3-30 kDa sheep jugular vein plasma ultrafiltrate. Anti-proliferative activity in vitro, in terms of the growth of MDA-MB231 breast cancers over 72h, smeared itself across a dozen fractions (Hart, 2103, Fig. 4 therein) and no candidate factor emerged. (Smearing also arose as an issue in physicochemical purifications. For example, anion exchange chromatography of rat hypothalamic extract revealed ~7+ kDa western gel bands across multiple fractions: Hart et al, 2017, Fig. 8 therein.) A sub-10 kDa filtrate of rat hypothalamus aqueous extract was purified via an affinity column using the goat polyclonal anti-EPL001 antibody and analysed with a 10-20 % tricine gradient gel under non-reducing conditions (Hart et al, 2017, p19). A Coomassie band corresponding to one at ~7 kDa seen by western blotting (op. cit., Fig. 10 therein) was subjected to Edman N-terminal sequencing (Applied Biosystems Procise cLC, at Bristol University, Bristol, UK). Data were heterogeneous even in what was the third attempt in this particular series, but subtracting kappa light chain supports the main inference as VVLTVNDGVQYNP (SEQ ID NO: 8). There are no credible bioinformatic hits to this sequence, N-terminal or otherwise, including when account is taken of subsidiary data options. The opening VVL looks like a reverberation of the Beale 4 second reading V/L (SEQ ID NO: 4), even more so when subsidiary possibilities are represented: V/LVLTV/L. There are EPL001 echoes in the form of LT and VxxxNN – the second N being a data option which in the form of **V**xxx**NNG** provided a correlation with NXPE family member 4 (Q5XI89, formerly FAM55D; Hart et al, 2017, pp20, 27 & 33). The sheep version of this aligns with six residues in ovine-derived EPL001, a 43% match, as follows: **GKV**TD**FNN**G. EPL001 is an N-terminal sequence, while this is not. NXPE family member 4 is secreted but a discordancy is that it is a basic protein with a predicted pI of 9.2 (Expasy get pI/Mw), whereas an acidic entity is indicated by behaviour in anion exchange chromatography. The doubleton QY in SEQ ID NO: 8 is potentially of interest. QY is also found in Harwell 1 (SEQ ID NO: 6), as well as in the 61-resdiue section of interest within SgII (as _188_QY_189_). A proteoform of SgII was eventually identified by Orbitrap MS as a candidate for the inhibitory factor in aqueous extract of rat hypothalamus, using the same goat antiserum as in the column purification but within an immunoprecipitation protocol (Hart et al, 2017).

**References**

Edman P (1950) Method for determination of the amino acid sequence in peptides. *Acta Chemica Scandinavica,* **4**, 283-293

Hart, JE (2001) Purified material having size and growth inhibitory effects on cells and tissues. Patent application: WO0193882.

Hart JE (2003) Isolated material having an antiorganotrophic effect. Patents granted to

Endocrine Pharmaceuticals Ltd, EP 1135145, 2003, US 7572465, 2009, and US7700132, 2010.

Hart, JE (2013) Proteinaceous compounds. US patent 8367801 granted to Endocrine Pharmaceuticals Ltd 2013; European patent EP 2234632 granted 2014.

Hart JE, Clarke IJ, Risbridger GP, Ferneyhough B, Vega-Hernandez M (2017) Mysterious inhibitory cell regulator investigated and found likely to be secretogranin II related. *PeerJ* 2017; **5**: e3833.

Smith, JB (2001) Peptide sequencing by Edman degradation. *Encyclopedia of Life Sciences*, Wiley Online Library; DOI: 10.1038/npg.els.0002688
