# Supplementary material for: Hexapeptides from mammalian inhibitory hormone hunt activate and inactivate nematode reproduction
Source: PLoS One. 2022 Dec 1;17(12):e0278049. doi: 10.1371/journal.pone.0278049 (PMC9714824; doi:10.1371/journal.pone.0278049)
Supplement: S3 File — Figshare: Species confirmation of the entomopathogenic nematode, Steinernema siamkayai, and pilot study. https://doi.org/10.6084/m9.figshare.16438062. This project contains data confirming the identity of the nematode species used and shows that the results of a pilot study of peptide administration were in line with those of the nematode experiment reported in the paper. (DOCX) [file pone.0278049.s003.docx]

**Supplementary Information 3 (S3)**

**Nematodes**

S3 is provided in support of ‘Hexapeptides from mammalian inhibitory hormone hunt activate and inactivate nematode reproduction’

**Species identification**

**ORGANISM** *Steinernema siamkayai*

Eukaryota; Metazoa; Ecdysozoa; Nematoda; Chromadorea; Rhabditida;

Tylenchina; Panagrolaimomorpha; Strongyloidoidea; Steinernematidae;

Steinernema.

**ACCESSION** MZ318695

***Steinernema siamkayai* isolate SM-01** internal transcribed spacer 1, partial sequence; 5.8S ribosomal RNA gene and internal transcribed spacer 2, complete sequence; and large subunit ribosomal RNA gene, partial sequence.

001 ggaaggatca ttattgagct aatttcttcc atttaatcag gtctttgctg tttgtctcta

061 agcattgact tgatcttgct ttgaatggtt tctagagacg tttggagcag tcatttaagc

121 gtgactgtga tgatgagcgt tttactttgc ttgcatttcg ctgtttcttg aatgcttagc

181 gatgagaatt aaagaggtct gctgactcgc catttttatt gataacaaaa acgttttgtt

241 ttgatttttg tgtcacttgt tgatgcatta ttcaattatc aagtcttatc ggtggatcac

301 tcggttcgta ggtcgatgaa aaacggggca aaaaccgtta tttggcgtga attgcagaca

361 tattgagcgc taaaattttg aacgcaaatg gcactaacag ggttatctgt tagtatgttc

421 aattgagggt cttttgacta gaatctggca atcggctgtg attgcttttt cggaaagtta

481 ttttgctttt tctaaagtga agtacctttt tggtatggct atttgattgt ctaatggatg

541 tctggttagc tgtttctttg ctagacgtct gcaatcattt ggctttgcgt agtgtttgaa

601 taataggtta gcgcgtttct tgctaactga cttttgcaca agcaagtgta atacgtttct

661 taaagtcagc ttttattcaa tttggttttc tgacttgatt tgtcggttta ctgtgctatg

721 ctttgtcaat cttttcgaac tagacctcaa tttgagcaag attacccgct gaacttaag

**PRIMERS**

Forward - TTGATTACGTCCCTGCCCTTT

Reverse - TTTCACTCGCCGTTACTAAGG

**S3 Table 1: Nematode pilot study fecundity data**

Mean number of A) Infective juveniles and B) Adults following inoculation of Petri dishes treated with EPL036 and EPL630 at 4 µL and 8 µL and an untreated control; Petri dishes were initially inoculated with 5 infective juveniles of *Steinernema siamkayai* following the method used in the main manuscript (Single way ANOVA presented beneath each data set).

**B) ADULTS**

**EPL036 EPL036 EPL630 EPL630**

**Reps Control 4 µL 8 µL µL 4 8 µL**

**________________________________________**

1 2 1 0 1 4

2 3 1 0 1 5

3 3 2 0 2 5

4 3 1 0 2 3

5 3 2 0 2 4

6 3 2 0 3 4

7 4 2 0 2 4

8 4 2 0 2 4

9 2 2 0 2 4

________________________________________

**Mean 3.0 1.7 0 1.9 4.1**

**ANOVA**

*Variation Between*

*Groups SS df MS F P-value F crit*

*85.422 4 21.356 72.528 8.65E-18 2.6059*

1. **INFECTIVE JUVENILES**

**EPL036 EPL036 EPL630 EPL630**

**Reps Control 4 µL 8 µL µL 4 8 µL**

**________________________________________**

1 32 56 87 5 5

2 25 55 67 6 2

3 30 34 70 14 3

4 45 42 74 14 3

5 38 54 79 9 5

6 23 34 80 13 5

7 36 37 66 12 6

8 34 36 74 5 6

9 26 41 85 13 8

________________________________________

**Mean 32.1 43.2 75.8 10.1 4.8**

**ANOVA**

*Variation Between*

*Groups SS df MS F P-value F crit*

*29298.7 4 7324.69 174.35 9.69E-25 2.6059*

**S3 Table 2: Nematode fecundity data**

Mean number of A) Infective juveniles and B) Adults following inoculation of Petri dishes treated with EPL036 and EPL630 at 4 µL and 8 µL and an untreated controls; Petri dishes were initially inoculated with either 500 or repeat with 1000 infective juveniles of *Steinernema siamkayai* following the method used in the main manuscript (Single way ANOVA presented beneath each data set).

1. **INOCULUM 500**

**A) INFECTIVE JUVENILES B) ADULTS**

**EPL036 EPL036 EPL630 EPL630**

| **ANOVA A INOCULUM 500** |  |  |  |  |  |  |
| --- | --- | --- | --- | --- | --- | --- |
| *Source of Variation* | *SS* | *df* | *MS* | *F* | *P-value* | *F crit* |
| Between Groups | 1.66E+08 | 5 | 33164789 | 15740.29 | 2.91E-12 | 4.387374 |
| Within Groups | 12642 | 6 | 2107 |  |  |  |
|  |  |  |  |  |  |  |
| Total | 1.66E+08 | 11 |  |  |  |  |
|  |  |  |  |  |  |  |

**Reps Control 4 µL 8 µL Control 4 µL 8 µL**

**_____________________________________________________**

1 3186 7080 9805 900 10 361

2 3131 7742 9534 894 10 361

3 3174 7623 9594 969 10 368

**_____________________________________________________**

**Mean 3164 7482 9644 921 10 363**

1. **INOCULUM 1000**
2. **INFECTIVE JUVENILES B) ADULTS**

**EPL036 EPL036 EPL630 EPL630**

| **ANOVA B INOCULUM 1000** |  |  |  |  |  |  |
| --- | --- | --- | --- | --- | --- | --- |
| *Source of Variation* | *SS* | *df* | *MS* | *F* | *P-value* | *F crit* |
| Between Groups | 9.44E+08 | 5 | 1.89E+08 | 698556.2 | 2.41E-32 | 3.105875 |
| Within Groups | 3244 | 12 | 270.3333 |  |  |  |
|  |  |  |  |  |  |  |
| Total | 9.44E+08 | 17 |  |  |  |  |
|  |  |  |  |  |  |  |

**Reps Control 4 µL 8 µL Control 4 µL 8 µL**

**________________________________________________________**

1 5493 13683 19141 1657 438 10

2 5526 13706 19149 1658 460 10

3 5523 13670 19193 1667 441 10

**________________________________________________________**

**Mean 5514 13686 19161 1661 446 10**
