# Supplementary material for: Hexapeptides from mammalian inhibitory hormone hunt activate and inactivate nematode reproduction
Source: PLoS One. 2022 Dec 1;17(12):e0278049. doi: 10.1371/journal.pone.0278049 (PMC9714824; doi:10.1371/journal.pone.0278049)
Supplement: S4 File — Figshare: Histological labelling of the ovine median eminence by an anti-EPL001 antibody and the effects of EPL001 and its scrambled-sequence control EPL030 on pituitary hormone release. https://doi.org/10.6084/m9.figshare.16438161. This project contains raw IHC images showing hypothalamic localisation, together with data on peptide effects on pituitary hormone release in vivo and in vitro. (DOCX) [file pone.0278049.s004.docx]

Supplementary Information 4 (S4)

Hypothalamus & Pituitary

S4 is provided in support of

‘Hexapeptides from mammalian inhibitory hormone hunt activate and inactivate nematode reproduction’

**Ovine median eminence**

**S4 Figure 1. Histological Depiction of the Ovine Median Eminence.**


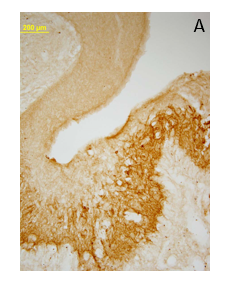


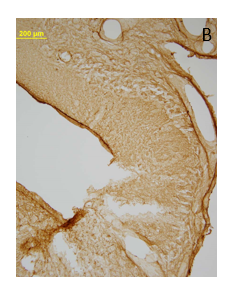


**S4 Figure 2. Labelling of the Ovine Median Eminence by Antibodies to EPL001.**

Immunohistochemical labelling of ovine median eminence sections by (A) rabbit polyclonal antibody ER87, dilution 1:1000 and (B) goat polyclonal antibody G530, dilution 1:1000. Both antibodies were raised to peptide EPL001


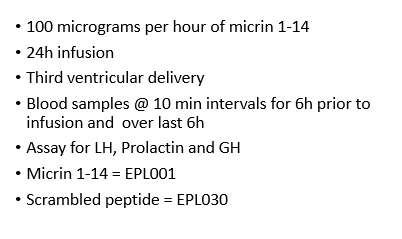


**S4 Figure 3. Experimental Design for the Intracerebroventricular Infusion of EPL001 and EPL030.**

Assay = RIA


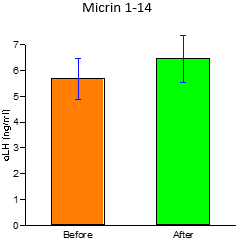

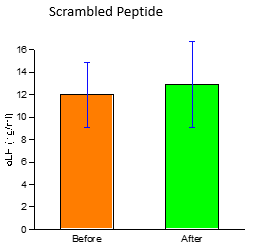


Micrin 1-14

Luteinising Hormone

Scrambled peptide

Before After

Before After

oLH (ng/ml)

oLH (ng/ml)

oLH = ovine luteinising hormone

**S4 Figure 4. Effects of Micrin 1-14 and Scrambled Peptide on Circulating Levels of ovine Luteinising Hormone.**

Peptides were given by intracerebroventicular infusion into one sheep each. Blood samples were taken at 10 min intervals Before and After infusion. See Materials and Methods for details. oLH = ovine luteinising hormone.


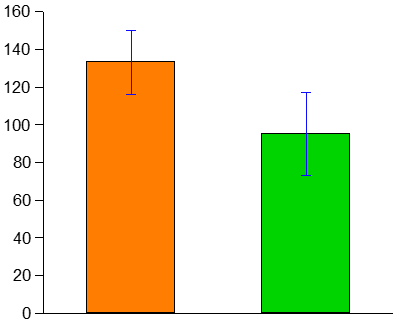

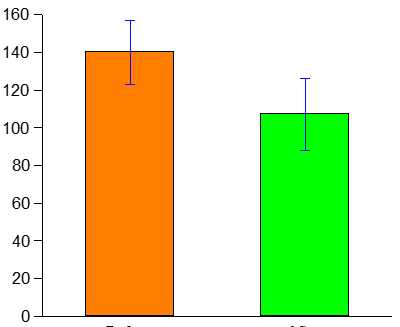


Micrin 1-14

Before After

Scrambled peptide

Before After

oPrl (ng/ml)

oPrl (ng/ml)

**S4 Figure 5. Effects of Micrin 1-14 and Scrambled Peptide on Circulating Levels of ovine Prolactin.**

Peptides were given by intracerebroventicular infusion into one sheep each. Blood samples were taken at 10 min intervals Before and After infusion. See Materials and Methods for details. oPrl = ovine prolactin


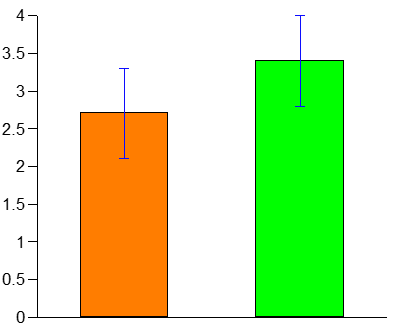


Micrin 1-14

Before After

Scrambled peptide


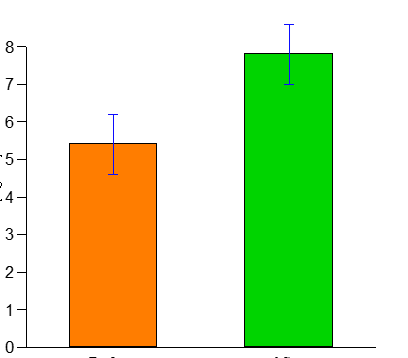


Scrambled peptide

Before After

oGH (ng/ml)

oGH (ng/ml)

**S4 Figure 6. Effects of Micrin 1-14 and Scrambled Peptide on Circulating Levels of ovine Growth Hormone.**

Peptides were given by intracerebroventicular infusion into one sheep each. Blood samples were taken at 10 min intervals Before and After infusion. See Materials and Methods for details. oPrl = ovine growth hormone

oPrl (ng/ml)

control

0.1 μg/ml micrin

1 μg/ml micrin

10 μg/ml micrin

**S4 Figure 7. Effects of Micrin 1-14 on Prolactin Secretion by Cultured Ovine Pituitary Cells.**

Ovine pituitary cells were grown in the presence of micrin 1-14 (0.1 – 10 μg/ml). Ovine prolactin (oPrl) secretion in the culture medium was measured by radioimmunoassay. The levels of secretion of hormones into medium gave concentrations that were greater than found in plasma. The culture medium was, therefore, diluted 1:25 in order to be read off the standard curve for the assay. The values on the Y axis are for the diluted samples. Data is the mean ± sem of determinations from six wells.

(see Wu et al 1997, J. Endocrinology 154:219-30 for ovine pituitary cell culture methodology)

Control

0.1 μg/ml micrin

1 μg/ml micrin

10 μg/ml micrin

+GnRH/GHRH/TRH

oPrl (ng/ml)

**S4 Figure 8. Effects of Micrin 1-14 on Prolactin Secretion by Cultured Ovine Pituitary Cells in the presence of GnRH/GHRH/TRH.**

Ovine pituitary cells were grown in the presence of micrin 1-14 (0.1 – 10 μg/ml). Ovine prolactin (oPrl) secretion in the culture medium was measured by radioimmunoassay. Other details as for Figure 7.

oPrl (ng/ml)

control

0.1 μg/ml scrambled

1 μg/ml scrambled

10 μg/ml scrambled

**S4 Figure 9. Effects of Scrambled Peptide (EPL030) on Prolactin Secretion by Cultured Ovine Pituitary Cells.**

Ovine pituitary cells were grown in the presence of EPL030 (0.1 – 10 μg/ml). Ovine prolactin (oPrl) secretion in the culture medium was measured by radioimmunoassay. Other details as for Figure 7.

oPrl (ng/ml)

0.1 μg/ml scrambled

1 μg/ml scrambled

10 μg/ml scrambled

+GnRH/GHRH/TRH

Control

**S4 Figure 10. Effects of Scrambled Peptide (EPL030) on Prolactin Secretion by Cultured Ovine Pituitary Cells in the presence of GnRH/GHRH/TRH.**

Ovine pituitary cells were grown in the presence of EPL030 (0.1 – 10 μg/ml). Ovine prolactin (oPrl) secretion in the culture medium was measured by radioimmunoassay. Other details as for Figure 7.

control

0.1 μg/ml micrin

1 μg/ml micrin

10 μg/ml micrin

oLH (ng/ml)

**S4 Figure 11. Effects of Micrin 1-14 on the Secretion of Luteinising Hormone by Cultured Ovine Pituitary Cells.**

Ovine pituitary cells were grown in the presence of micrin 1-14 (0.1 – 10 μg/ml). Ovine luteinising hormone (oLH) secretion in the culture medium was measured by radioimmunoassay. Other details as for Figure 7.

Control

0.1 μg/ml micrin

1 μg/ml micrin

10 μg/ml micrin

+GnRH/GHRH/TRH

oLH (ng/ml)

**S4 Figure 12. Effects of Micrin 1-14 on the Secretion of Luteinising Hormone by Cultured Ovine Pituitary Cells in the presence of GnRH/GHRH/TRH.**

Ovine pituitary cells were grown in the presence of micrin 1-14 (0.1 – 10 μg/ml). Ovine luteinising hormone (oLH) secretion in the culture medium was measured by radioimmunoassay. Other details as for Figure 7.

oLH (ng/ml)

control

0.1 μg/ml scrambled

1 μg/ml scrambled

10 μg/ml scrambled

oLH (ng/ml)

control

0.1 μg/ml scrambled

1 μg/ml scrambled

10 μg/ml scrambled

**S4 Figure 13. Effects of Scrambled Peptide (EPL030) on Luteinising Hormone Secretion by Cultured Ovine Pituitary Cells.**

Ovine pituitary cells were grown in the presence of EPL030 (0.1 – 10 μg/ml). Ovine luteinising hormone (oLH) (oPrl) secretion in the culture medium was measured by radioimmunoassay. Other details as for Figure 7.

oLH (ng/ml)

0.1 μg/ml scrambled

1 μg/ml scrambled

10 μg/ml scrambled

+GnRH/GHRH/TRH

Control

**S4 Figure 14. Effects of Scrambled Peptide (EPL030) on the Secretion of Luteinising Hormone by Cultured Ovine Pituitary Cells in the presence of GnRH/GHRH/TRH.**

Ovine pituitary cells were grown in the presence of EPL030 (0.1 – 10 μg/ml). Ovine luteinising hormone (oLH) secretion in the culture medium was measured by radioimmunoassay. Other details as for Figure 7.

oLH (ng/ml)

oLH (ng/ml)

+GnRH/GHRH/TRH

Control 0.1 μg/ml 1 μg/ml 10 μg/ml

scrambled scrambled scrambled

oLH (ng/ml)

Control 0.1 μg/ml 1 μg/ml 10 μg/ml

scrambled scrambled scrambled

+GnRH/GHRH/TRH

oLH (ng/ml)

Control 0.1 μg/ml 1 μg/ml 10 μg/ml

micrin micrin micrin

Control 0.1 μg/ml 1 μg/ml 10 μg/ml

micrin micrin micrin

Figure fffff

**S4 Figure 15. Effects of EPL001 and EPL030 on luteinising hormone secretion by ovine pituitary cells.**

Ovine pituitary cells were grown in the presence of micrin 1-14 (EPL001 – top panel) or scrambled peptide (EPL030 – lower panel) (0.1 – 10 μg/ml). in the absence or presence of GnRH/GHRH/TRH. Ovine luteinising hormone (oLH) secretion in the culture medium was measured by radioimmunoassay. Other details as for Figure 7 apart from a 1:50 dilution of culture medium was used in the radioimmunoassay.

oLH (ng/ml)

oLH (ng/ml)

Control

0.1 μg/ml micrin

1 μg/ml micrin

10 μg/ml micrin

Control

0.1 μg/ml micrin

1 μg/ml micrin

10 μg/ml micrin

+GnRH/GHRH/TRH

oLH (ng/ml)

oLH (ng/ml)

Control

0.1 μg/ml scrambled

1 μg/ml scrambled

10 μg/ml scrambled

10 μg/ml scrambled

Control

1 μg/ml scrambled

0.1 μg/ml scrambled

+GnRH/GHRH/TRH

**S4 Figure 16. Effects of EPL001 and EPL030 on luteinising hormone secretion by ovine pituitary cells.**

Ovine pituitary cells were grown in the presence of micrin 1-14 (EPL001 – top panel) or scrambled peptide (EPL030 – lower panel) (0.1 – 10 μg/ml). in the absence or presence of GnRH/GHRH/TRH. Ovine luteinising hormone (oLH) secretion in the culture medium was measured by radioimmunoassay. Other details as for Figure 7 apart from a 1:50 dilution of culture medium was used in the radioimmunoassay.
