# Supplementary material for: Hexapeptides from mammalian inhibitory hormone hunt activate and inactivate nematode reproduction
Source: PLoS One. 2022 Dec 1;17(12):e0278049. doi: 10.1371/journal.pone.0278049 (PMC9714824; doi:10.1371/journal.pone.0278049)
Supplement: S5 File — Figshare: The toxicity and efficacy of EPL001 and its scrambled-sequence control EPL030 in immunodeficient mice. https://doi.org/10.6084/m9.figshare.16438338. This project presents data showing the lack of toxicity and efficacy of the peptides in immunodeficient mice. (DOCX) [file pone.0278049.s005.docx]

**Supplementary Information 5 (S5)**

**Murine Studies**

S5 is provided in support of ‘Hexapeptides from mammalian inhibitory hormone hunt activate and inactivate nematode reproduction’

**1. Evaluation of acute toxicity of EPL001 and EPL030 in immunodeficient mice**

For toxicity studies female Balb/c immunodeficient nude mice were used (B & K Universal, Hull, UK), aged 6-8 weeks old. They were housed in cages in isolation cabinets in an air-conditioned room with regular alternating cycles of light and darkness and received Teklad 2018 (Envigo) diet and water *ad libitum*. EPL001 and EPL030 in PBS at pH7.4 were administered to groups of 2 mice intraperitoneally (i.p.) daily on days 0-4 at 500mg/kg/dose and then 1000mg/kg/dose. Following treatment, body weight was measured on a regular basis, and behaviour and general appearance monitored visually to assess for deleterious effects (e.g. dehydration, impaired mobility, hunched posture, low body temperature, ulceration and significant body weight loss), with any effects during the study recorded. If body weight loss was >15% over a 72-hour period or if animal behaviour and appearance were significantly altered, then mice were immediately sacrificed by cervical dislocation. **S4 Figure 1**, below, shows the bodyweight curves for the 2 dose levels.


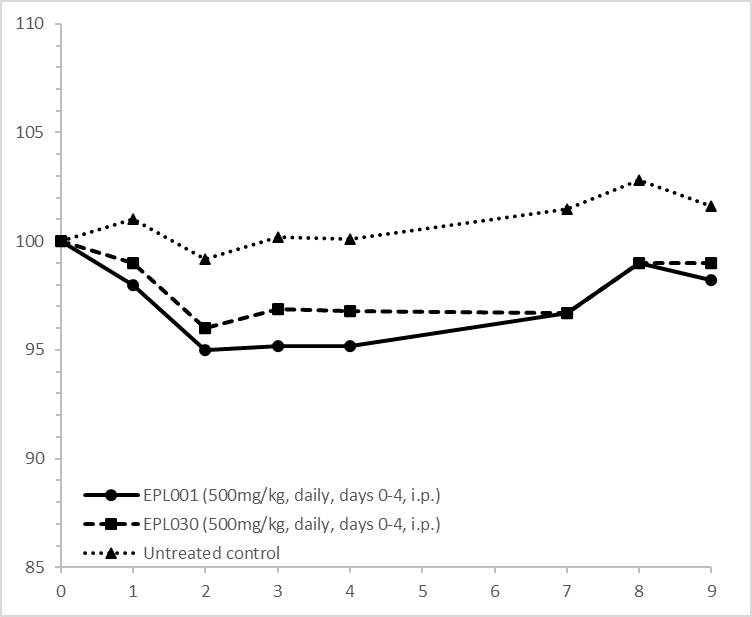

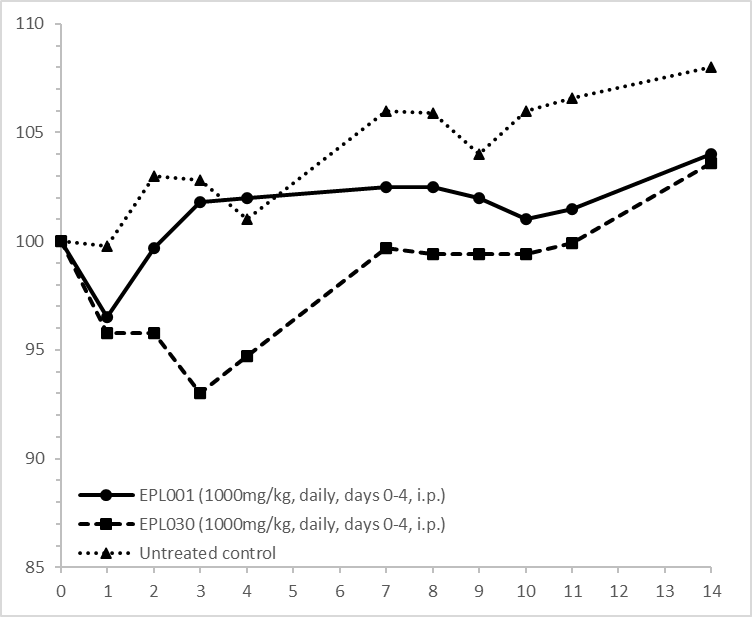


Both EPL001 and EPL030 were well-tolerated at these relatively high doses, with the slight decreases seen in weight loss within normal physiological levels (maximum weight loss occurred at day 2, with 5.0% loss for EPL001 and 4.0% loss for EPL030 for the 500mg/kg schedule, and 3.5% loss on day 1 for EPL001, and 7.0% on day 3 for EPL030 for the 1000mg/kg schedule), with a return to normal weight levels after 7 days. No other discernible signs of toxicity were evident for both molecules at both dose ranges.

**2. Evaluation of the efficacy of EPL001 and EPL030, and a monoclonal antibody to EPL001 in immunodeficient mice bearing MCF-7 human breast adenocarcinoma xenografts**

The MCF-7 human breast adenocarcinoma model was selected for two xenograft studies. An oestrogen pellet was implanted subcutaneously in the dorsal area 24 hours prior to 2-3mm^3^ fragments of MCF-7 tumour taken from donor tumours transplanted subcutaneously in the abdominal flanks of the efficacy study mice. Once tumour volumes reached approximately 32 mm^3^ (as measured by calipers, designated treatment day 0) mice were randomised into treatment groups (n=8).

In the first study four groups were used as follows: EPL001 or EPL030 at 100mg/kg/day administered i.p. on days 0, 1, 4, 5, 6, 7, 8, 11, 12, 13, 14, 15, 18, 19 and 20, plus a mouse anti-EPL001 monoclonal antibody or a control antibody directed at mouse haemocytes at 4mg/kg/day administered i.p. on days 0, 1, 2, 3, 4, 7, 8, 9, 10, 11, 14, 15, 16, 17 and 18 .

In the second study three groups were used as follows: EPL001 or EPL030 at 100mg/kg/day administered i.p. on days 0-17, plus an untreated control group.

Tumour volume, using calipers, and animal body weight were recorded throughout the experiment and normalised to the respective volume on the initial day of treatment (day 0). Mann-Whitney U tests were conducted to determine the statistical significance of any differences in growth rate (based on tumour volume doubling time) between control and treated groups.

**S4 Figure 2**, below, and **S4 Table 1** on the next page show the tumour growth curves for a) the peptide groups (left), and b) the antibody groups (right) demonstrating no significant delay in tumour growth for EPL001 versus EPL030, and the anti-EPL001 antibody versus the control antibody.


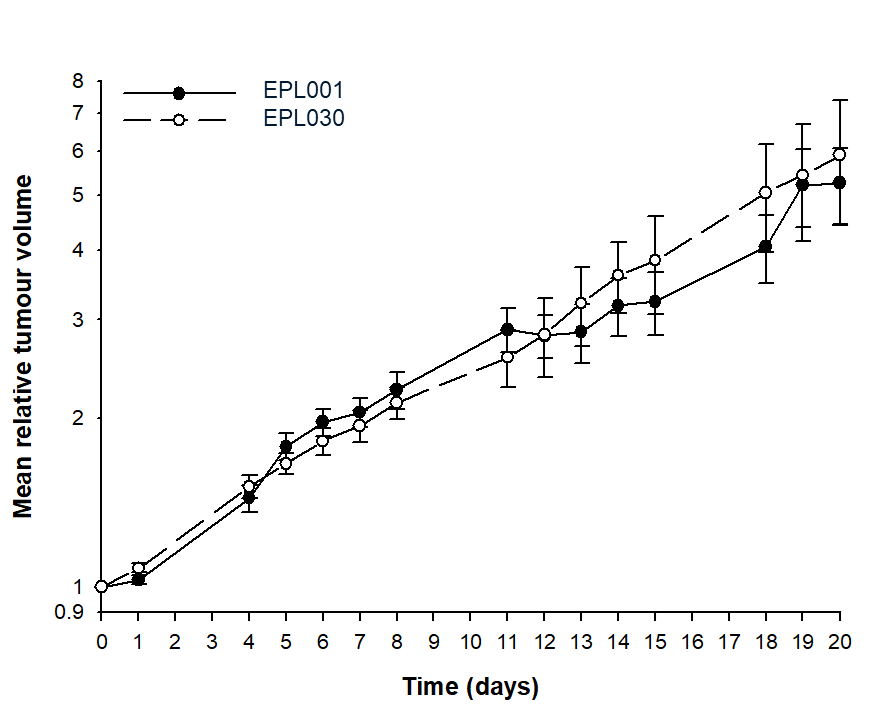

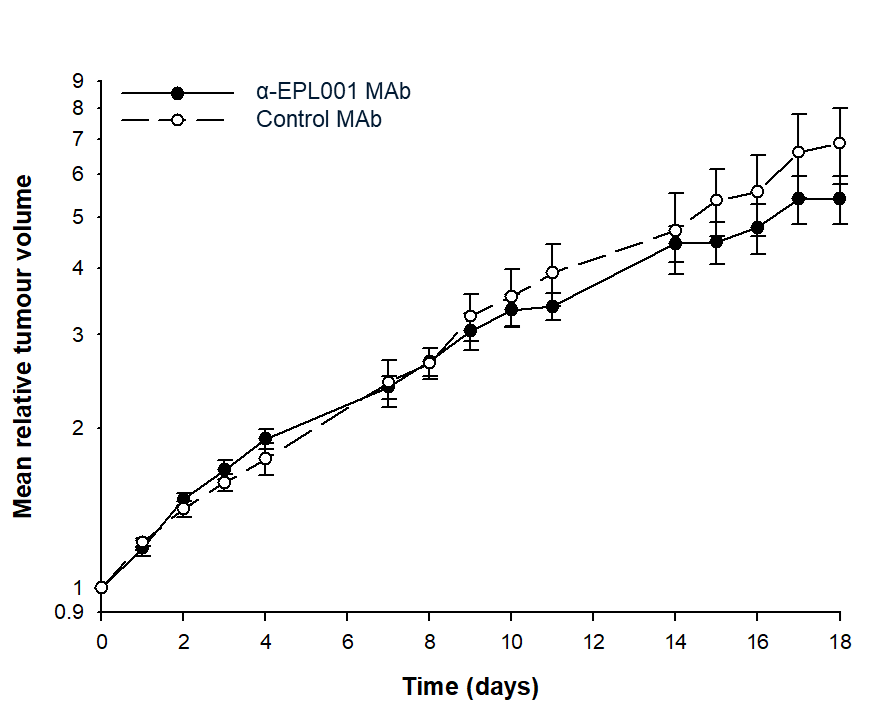


| Group | Median RTV2 (days) | Growth delay (days) | Signif. | Max % weight loss |  | Group | Median RTV2 (days) | Growth delay (days) | Signif. | Max % weight loss |
| --- | --- | --- | --- | --- | --- | --- | --- | --- | --- | --- |
| EPL001 | 7.7 | 0.4 | p>0.05 NS | 3.6 |  | α-EPL001 mAb | 4.1 | 1.8 | p>0.05 NS | 2.1 |
| EPL030 | 7.3 | - | - | 1.2 |  | Control mAb | 5.9 | - | - | 3.9 |

**S5 Figure 3** and **S5 Table 2**, both below, show that no statistically significant tumour growth delay was seen with either EPL001 or EPL030 compared to the control.


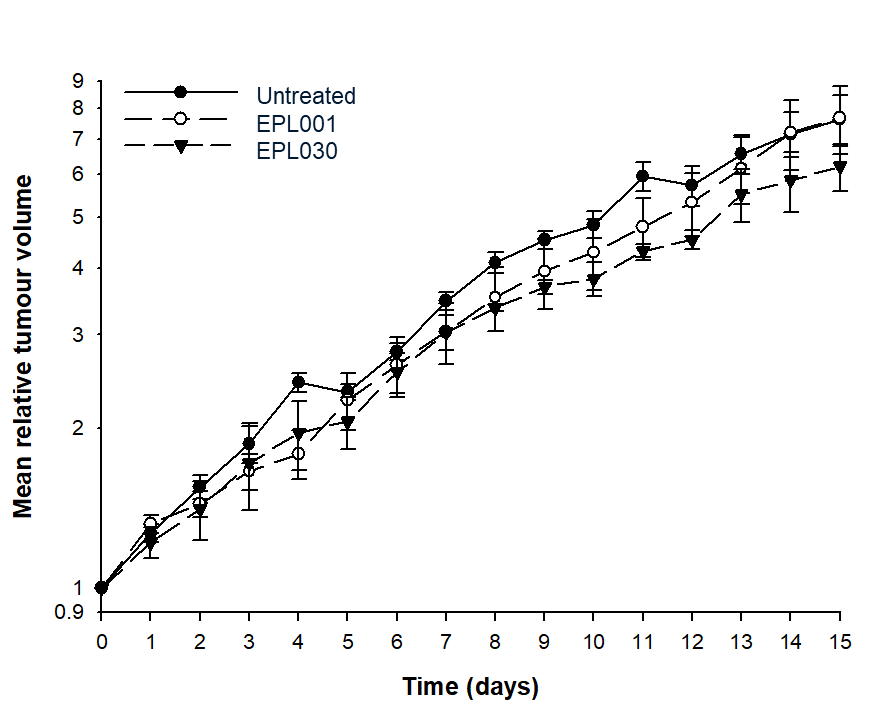


| Group | Median RTV2 (days) | Growth delay (days) | Signif. | Max % weight loss |
| --- | --- | --- | --- | --- |
| EPL001 | 4.6 | 1.1 | p>0.05 NS | 0 |
| EPL030 | 3.6 | 0.1 | p>0.05 NS | 2.4 |
| Untreated | 3.5 | - | - | 2.5 |
