# Supplementary material for: Hexapeptides from mammalian inhibitory hormone hunt activate and inactivate nematode reproduction
Source: PLoS One. 2022 Dec 1;17(12):e0278049. doi: 10.1371/journal.pone.0278049 (PMC9714824; doi:10.1371/journal.pone.0278049)
Supplement: S6 File — Figshare: The effects of EPL001 on stem cell proliferation, phenotype and gene expression. https://doi.org/10.6084/m9.figshare.16438392. This project presents data showing that EPL001 reduces proliferation and influences gene expression without changing stem cell phenotype. (DOCX) [file pone.0278049.s006.docx]

**Supplementary Information 6 (S6)**

**Peptide Immobilisation and Gene Studies**

S6 is provided in support of ‘Hexapeptides from mammalian inhibitory hormone hunt activate and inactivate nematode reproduction’


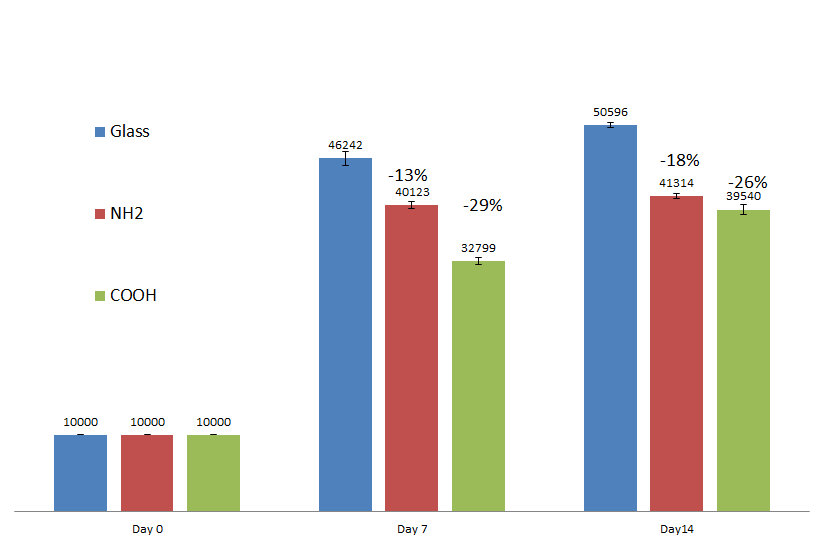


**S6 Figure 1. The Effects of EPL001 Immobilisation Binding Options on the Proliferation of Human Marrow Derived Mesenchymal Stem Cells.**

Human marrow derived mesenchymal stem cells (hMSCs) were immobilised onto glass coverslips at 0.01 mmol/cm^2^, utilising NH or COOH binding options for the synthetic 14mer peptide EPL001. Cell numbers were determined by CyQuant®.

NH2 = EPL001 tethered by its N terminus

COOH = EPL001 tethered by its C terminus


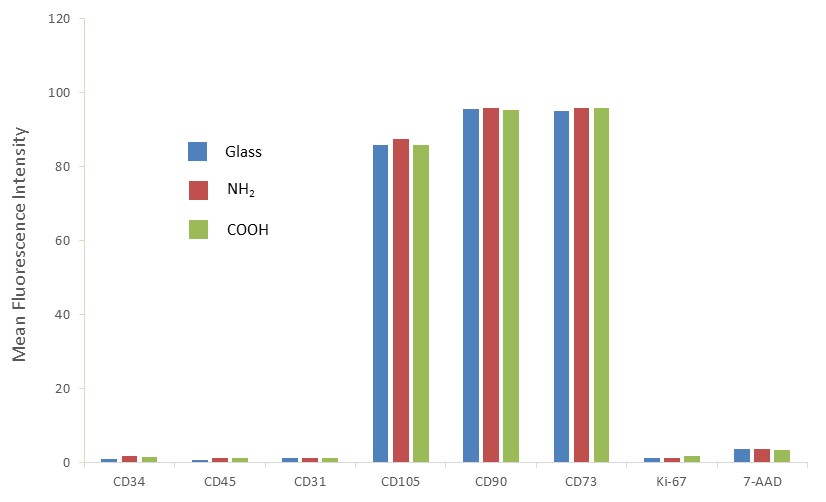


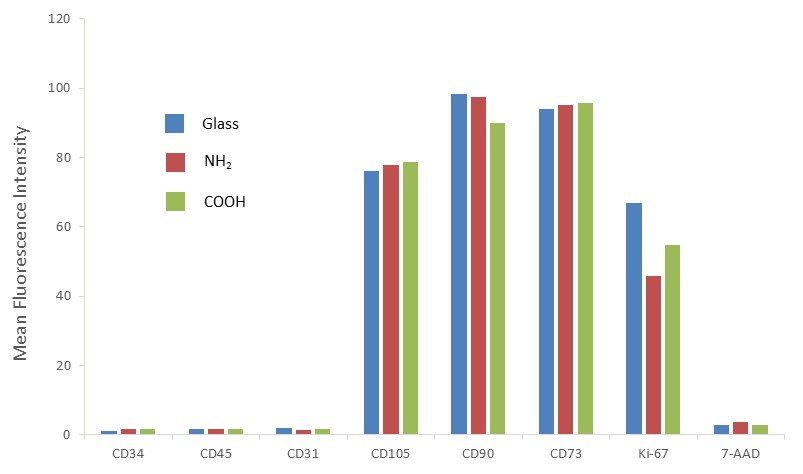


**S6 Figure 2. FACS Analysis of Stem Cell Markers on hMSCs Cultured in the Presence of EPL001.**

Peptide EPL001 was immobilised onto glass coverslips at a concentration of 10μmoles/cm^2^ as described in Materials and Methods. Coverslips were cultured with hMSCs in the wells of a 24-well plate. Viability (7-AAD FACS), proliferation (Ki67 FACS), cell cycle (Propidium Iodide FACS), Stem cell phenotype (antigen expression FACS: CD34, CD45, CD31, CD105, CD90 and CD73). Determinations were at 7 days (top panel) and 14 days (lower panel).


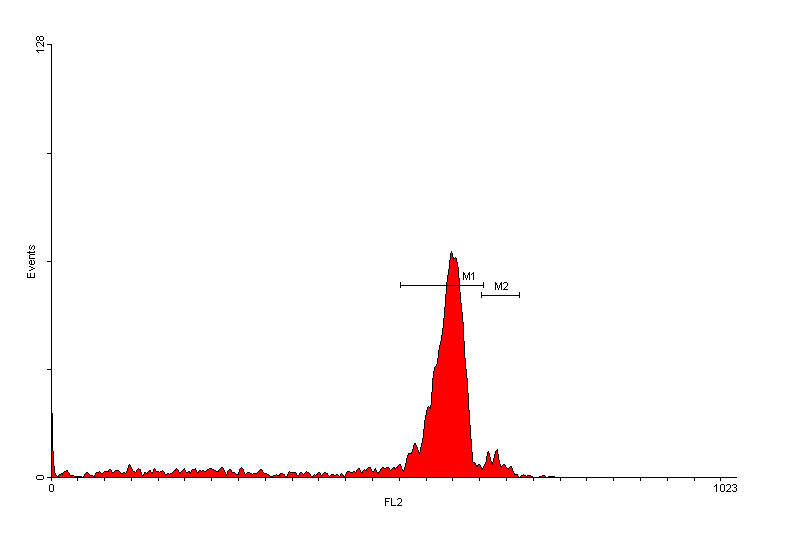
aa a

A


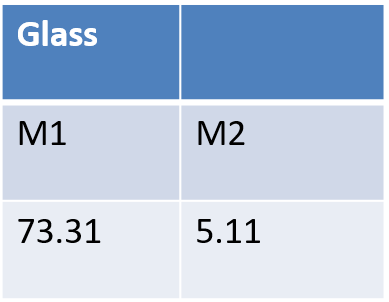


M1 G0/G1

M2 S/G2


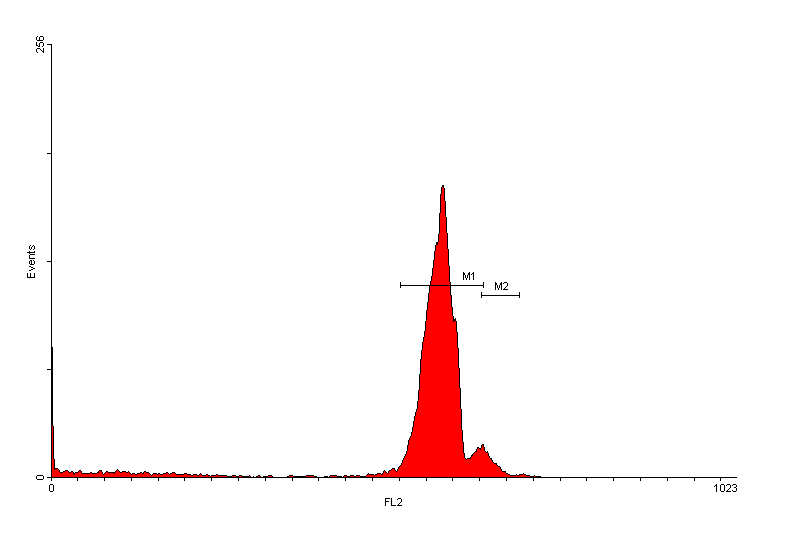


B

a

M1 G0/G1

M2 S/G2


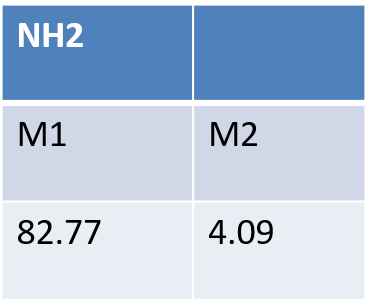


C


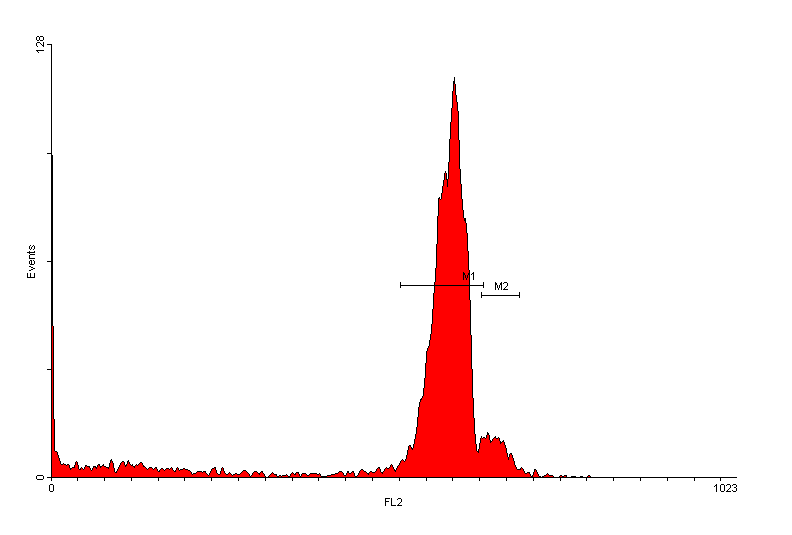


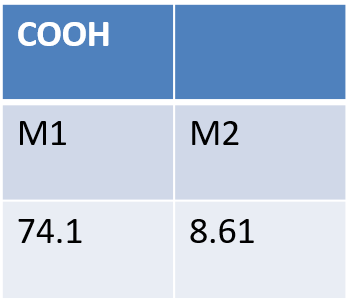


M1 G0/G1

M2 S/G2

**S6 Figure 3. Effects of EPL001 Tethering Options on Cell Cycle Phases.**

Peptide EPL001 was immobilised onto glass coverslips at a concentration of 10μmoles/cm^2^ as described in Materials and Methods. Coverslips were cultured with hMSCs in the wells of a 24-well plate. Growth was determined by PI and Ki-67 staining.

| a) | EPL001 | EPL140 |
| --- | --- | --- |
| *Bmal1* | ↑↑ | ↑ |
| *Per2* | ↓ | ↓ |
| *Rev-ErbA* | - | ↓ |
| *OCN* | ↓ | ↓ |
| *Sox9* | ↓ | ↓↓ |
| *PPARγ* | ↓ | ↓↓ |

| b) | EPL001 | EPL140 |
| --- | --- | --- |
| *Bmal1* | ↑ | ↑↑ |
| *Per2* | ↑ | ↑↑ |
| *Rev-ErbA* | ↓ | ↓ |
| *OCN* | ↓↓ | ↓↓ |
| *Sox9* | (↓) | (↑) |
| *PPARγ* | ↓↓ | ↓ |

**S6 Figure 4. Effects of the 14mers EPL001 and EPL140 on gene expression in cultured cells.**

(a) (hMSCs) and (b) hDPSC (Human Dental Pulp Derived Mesenchymal-Like Stem/Progenitor Cells) were treated with peptides EPL001 (0.15μM) or EPL140 (15μM) for three days.

**Reference**

Rogers E (2018) Exploring the Potential of the Physio-Mechanical Environment and Circadian Timing in Adult Progenitor Cell Differentiation. PhD Thesis, University of Liverpool, Liverpool, UK, pp144-181. doi: [10.17638/03044424](https://eur03.safelinks.protection.outlook.com/?url=http%3A%2F%2Fdoi.org%2F10.17638%2F03044424&data=04%7C01%7Cdavid.howlett%40kcl.ac.uk%7C111f329eae0b4c0fa12c08d8efabcf50%7C8370cf1416f34c16b83c724071654356%7C0%7C0%7C637522868481326188%7CUnknown%7CTWFpbGZsb3d8eyJWIjoiMC4wLjAwMDAiLCJQIjoiV2luMzIiLCJBTiI6Ik1haWwiLCJXVCI6Mn0%3D%7C1000&sdata=gNhC9z60%2BYQ4bYLQryYpb9jiqsy3e5aGrwnPR5iTsN8%3D&reserved=0). **Note: In this source ‘EPL’ is used in two senses. The sense relevant here is in regard to the chemically synthesized proprietary 14mer peptides EPL001, EPL140 & EPL142 of the present paper’s Table 1, where EPL stands for Endocrine Pharmaceuticals Limited.**

EPL001 alanine substitution study – see Figures 5 and 6 below.

Reference:

Pu FR, Chen R, Hart JE and Hunt JA. Induction of populations of quiescent, slow-cycling bone marrow mesenchymal stem cells using substrate tethered peptides. Abstract published in Proceedings of the 9^th^ World Biomaterials Congress 2012, China; Curran Associates, NY, 2014 (4 volumes); ISBN: 978-1-62993-314-6. (Conference poster, reproduced below, with Figure numbers adjusted for the present file.)

Induction of populations of quiescent, slow-cycling bone marrow mesenchymal stem cells using substrate-tethered peptides

**Fanrong Pu^#^, Rui Chen^#^, John E Hart^*^ and John A Hunt^#^**

*^#^Clinical Engineering,UKCTE, Institute of Ageing and Chronic Disease, University of Liverpool, Duncan Building, Daulby Street, Liverpool, UK. Fax: 0044 151 706 4915; Tel: 0044 151 706 5920; E-mail:* [*frpu@liv.ac.uk*](mailto:frpu@liv.ac.uk)

*^*^Endocrine Pharmaceuticals Ltd, Tadley, Hampshire, RG26 3TA, UK*

**Introduction**

Adult stem cells are maintained in a quiescent state but are able to exit quiescence and rapidly expand and differentiate in response to stress. The quiescent state appears to be necessary for preserving the self-renewal of stem cells and is a critical factor in the resistance of cancer stem cells (CSCs) to chemotherapy and targeted therapies. Thus, an improved understanding of the molecular mechanisms of quiescence in adult stem cells is critical for the development of molecularly targeted therapies against quiescent CSCs in different cancers. In the present study, the mild inhibitor EPL001^1^ and alanine-substituted derivative peptides were chemically synthesised and the activity examined *in vitro* of the resulting populations of quiescent, slow-cycling bone marrow mesenchymal stem cells (BMSCs).

**Materials & Methods**

Four 14-aa peptides were synthesised: MKPLTGKVKEFNNI (EPL001), AAAAAGKVKEFNNI (EPL590), MKPLTAAAAEFNNI (EPL545) and MKPLTGKVKEAAAA (EPL104). The peptides were generated from l-isomer amino acids by solid phase peptide synthesis (Model 9050, Milligen). All peptides were N-terminally tethered to the substrate by a bioconjugate technique. MSCs were derived from human bone marrow and maintained in DMEM with 10% FSC. Cells were seeded on peptide treated surfaces including a glass control in a 24-well plate with cell density 5 x 10^3^. The growth inhibitory activity was examined via cell proliferation assays and cell cycle analysis, using PI and Ki-67 staining. The cell phenotype and viability (7-AAD) were analysed by flow cytometry.

**Results & Discussion, and Conclusion***

Mean cells numbers were lower in all the peptide exposed groups than controls (‘Glass’) (Fig.5b). Only the result for EPL104 reached statistical significance, a finding in line with morphological observations (Fig 5a). The number of cells in the G0/G1 phase for EPL104 was 90% compared with 86% for glass control (Fig.6a), with correspondingly fewer cells in the S and G2/M phases (Fig.6b). No differences in cell cycle analysis (Fig 6c) or phenotype (Fig 6d) between the groups were detected by FACS. Overall, alanine substitution did little to further the characterisation of these molecules, but although the inhibition showed by these particular peptides was weak, slow to assert itself and possibly involved an unhelpfully non-specific effect, the general principle of using substrate-tethered molecules to obtain quiescent BMSCs is here demonstrated.

**Reference**

1: J L Haylor *et al*, *Regulatory Peptides*, **152**, 48-53, 2009


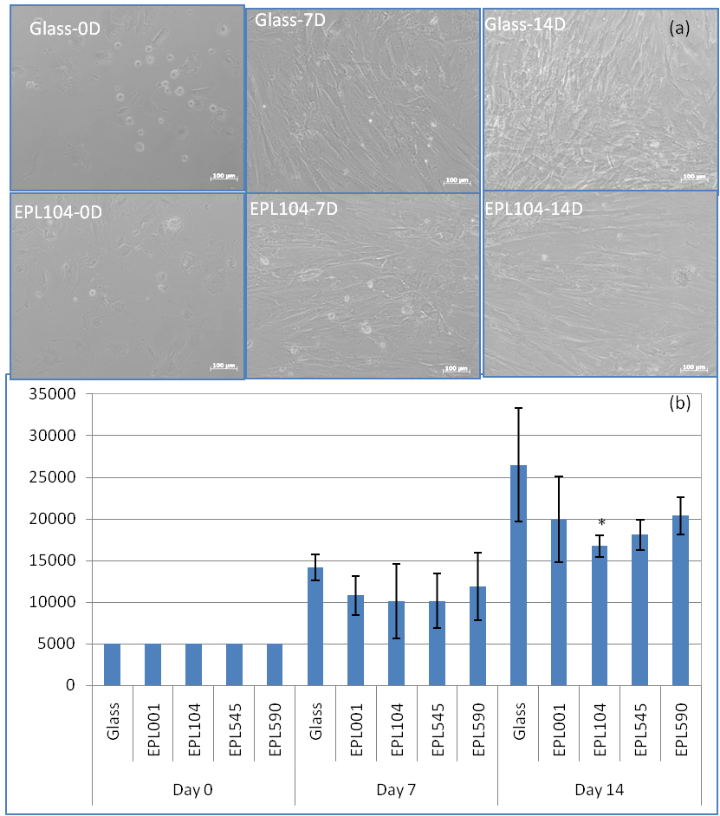


**S6 Figure 5. The Effects of Alanine-Substitution within EPL001 on the Proliferation of hMSCs grown on peptide-tethered glass coverslips.**

hMSCs were studied after 3 hours (Day 0), 7 and 14 days proliferation on (a) EPL104 treated or glass control coverslips or (b) a range of alanine-substituted versions of EPL001 with proliferation being determined by CyQuant® assay (* *P* < 0.05 compared to control glass surface, N=3).


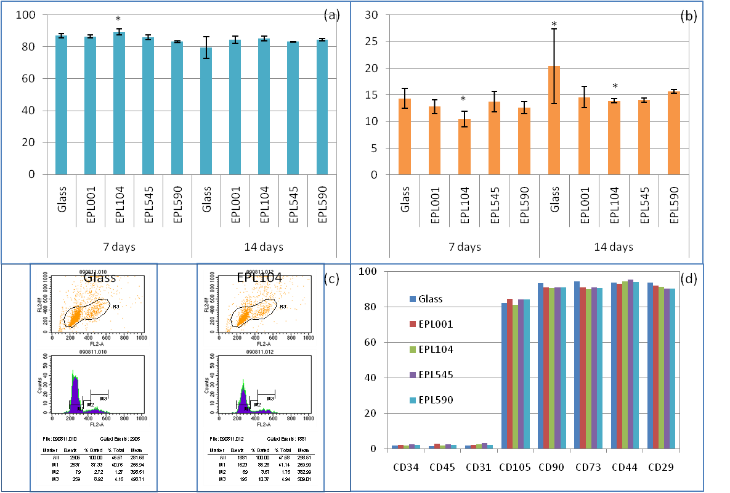


**S6 Figure 6. Cell cycle and phenotype analysis of hMSCs cultured on peptide treated surfaces for 7 and 14 days.**

(a) cell cycle distribution in G0/G1(% of total); (b) in S and G2/M phases (* *P* < 0.05 compared to control glass surface, N=3); (c) FACS cell cycle analysis of hMSC at 14 days of culture; (d) cell phenotype by FACS.
