# Supplementary material for: Hexapeptides from mammalian inhibitory hormone hunt activate and inactivate nematode reproduction
Source: PLoS One. 2022 Dec 1;17(12):e0278049. doi: 10.1371/journal.pone.0278049 (PMC9714824; doi:10.1371/journal.pone.0278049)
Supplement: S8 File — Figshare: Proposed ‘route’ taken by Edman sequencing to provide EPL001 from sSgII-70. https://doi.org/10.6084/m9.figshare.16438452. This project explores the concept of Edman Nonsequentalism, whereby chemical analysis is deemed to have misread the amino acid sequence of the deduced endogenous factor sSgII-70. (DOCX) [file pone.0278049.s008.docx]

**Supplementary Information 8 (S8)**

**Edman Nonsequentialism**

S8 is provided in support of ‘Hexapeptides from mammalian inhibitory hormone hunt activate and inactivate nematode reproduction’

Edman sSgII-14 α amines

cycle (=)

0 =M L K T G E K P V F K •N N I 1

1 (**M**) =L K T G E =K P V F K •N N I 2

2 (M) =L K T G E (**K**) =P V F K •N N I 2

3 (M) =L K T G E (K) (**P**) =V F K •N N I 2

4 (M) (**L**) =K =T G E (K) (P) =V F K •N N I 3

5 (M) (L) =K (**T**) =G E (K) (P) =V F K •N N I 3

6 (M) (L) =K (T) (**G**) =E (K) (P) =V F K •N N I 3

7 (M) (L) (**K**) (T) (G) =E (K) (P) =V F K •N N I 3

8 (M) (L) (K) (T) (G) =E (K) (P) (**V**) =F =K •N N I 3

9 (M) (L) (K) (T) (G) =E (K) (P) (V) =F (**K**) •N N I 2

10 (M) (L) (K) (T) (G) (**E**) (K) (P) (V) =F (K) •N N I 2

11 (M) (L) (K) (T) (G) (E) (K) (P) (V) (**F**) (K) •=N N I 1

12 (M) (L) (K) (T) (G) (E) (K) (P) (V) (F) (K) •(**N**) =N I 1

13 (M) (L) (K) (T) (G) (E) (K) (P) (V) (F) (K) •(N) (**N**) =I 1

14 (M) (L) (K) (T) (G) (E) (K) (P) (V) (F) (K) (N) (N) (**I**) 0

**S8 Figure 1.** Tabular representation of the 14-cycle Edman sequencing process by which EPL001 can be obtained from sSgII-14. The non-contiguity of the C-terminal NNI with the first eleven N-terminal residues is indicated by a dot (•). Residues removed are in brackets. The emphasized bracketed residue is the one taken at each cycle, yielding the zigzag reading path for EPL001 of the paper’s Fig. 1 sequence grid. Available α amines are indicated (=) at the end of each cycle. These free amines are deduced from the residue just taken and the one taken next, according to the sequence of EPL001.

M L K T G E K P V F K N N I

1 4 7 5 6 10 2 3 8 11 9 12 13 14

-5

+6

-6

+6

+

+1

+1

+1

+2

+4

+2

-2

+1

**S8 Figure 2.** Proposed route of Edman sequencing of sSgII-70 to provide EPL001. The non-contiguity of the C-terminal NNI with the first eleven N-terminal residues is indicated by a dot (•). To obtain EPL001 there are five major moves along the aa chain, towards and away from the C terminus, and eight minor moves, mostly towards the C terminus. A spiral is connoted.


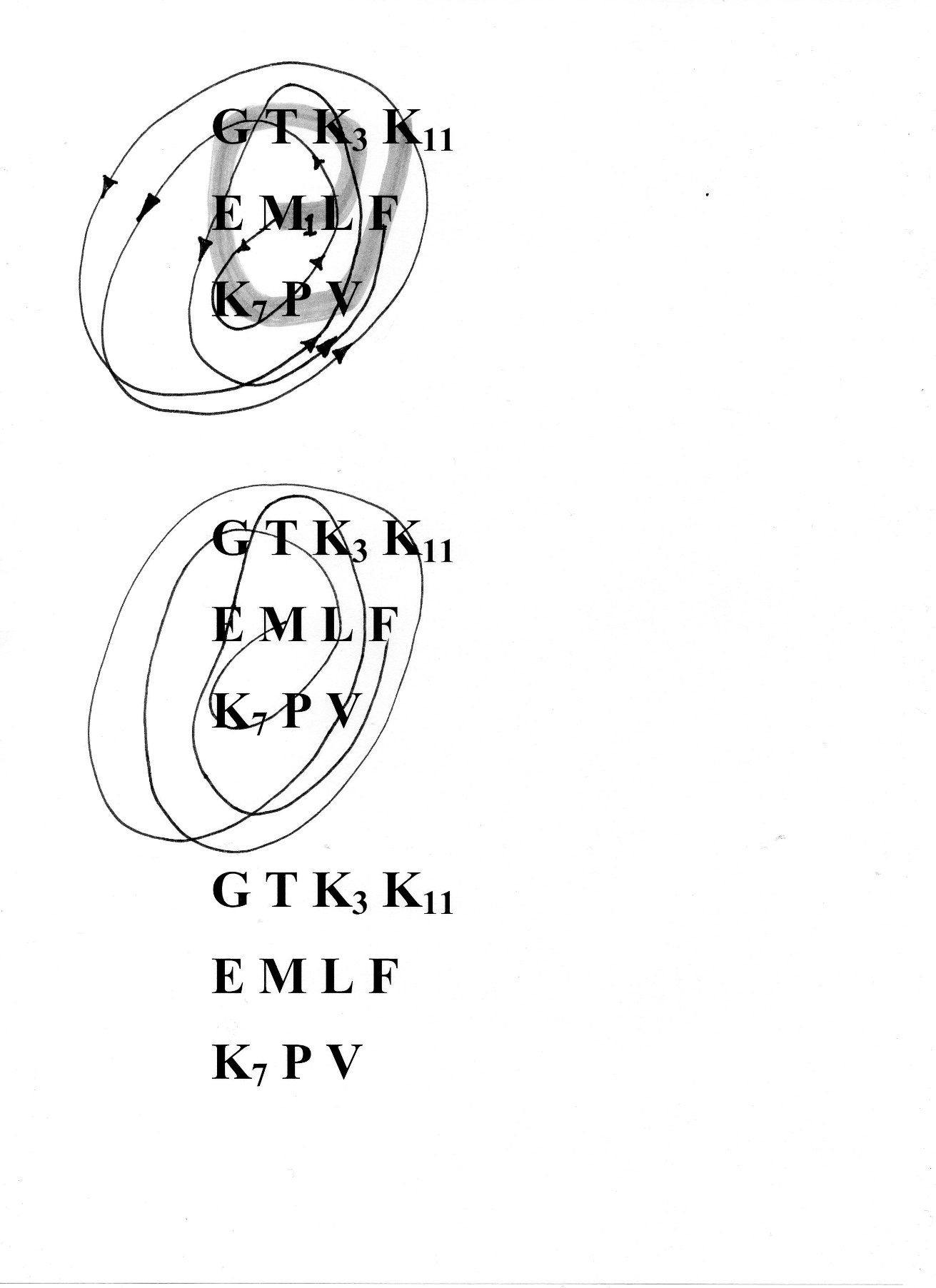


**S8 Figure 3.** **Double spiral stylisation.** The aa sequence of sSgII-11 (MLKTGEKPVFK) is given by the shaded track and the conjectured path of the equivalent Edman EPL001 sequence (MKPLTGKVKEF) as an arrowed line. A double spiral with methionine as the shared origin can be a topological heuristic device to address the question of accessibility. L2 should be taken second after M1 but appears fourth in EPL001. Why? Because of steric hindrance by P8. Once P8 is out of the way L2 is read.

M L K_3_ T G E K_7_ P V F K_11_ N N I

**S8 Figure 4**. Transforming sSgII-14 into EPL001 requires the movement of only five sSgII-14 residues, four charged and one in a class of its own: K3**^+^**, K7**^+^**, K11**^+^**, E6**^-^** and P8 (unique in being a proteinogenic *secondary* amino acid). The probability of choosing these five residues is about 1 in 2000 (S2). This analysis supports the view that something puzzling went on with the Edman chemical sequencing, relating at least in part to charged residues.

The paper explores as meaningful anagrams MLKTGEKPV∙FK∙NNI (sSgII-14) and MKPLTGKVKEFNNI (EPL001), yet how can they be? It is asserted that the residues in the three endogenous sSgII sequence modules were read in the wrong order by the Edman machine, instead of one by one from the N terminus, to give the EPL001 readout (SEQ ID NO: 3 of S1). This is the concept of Edman Nonsequentialism. Given the seven variable ovine sequences obtained (S1), Edman Nonsequentialism has the status of an observation not a hypothesis. Why might residues have been read in the wrong order? Speculating, because of the aberrant availability of free amine groups due to out-of-sequence peptide bond cleavages amounting to a multistage (non-enzymatic) depolymerisation. These bond breakages were ultimately due to the molecular characteristics of the sSgII proteoform sSgII-70, deduced to be a crosslinked spiral with a C terminus looping back to be bound near the N terminus. Polyanionic motifs in SgII-70 contribute to a picture of structural deformation onto the reaction membrane, with the potential for steric hindrance of the Edman reagent. Seemingly important in the reading order are charged residues (see below). Such residues even seem important by omission, with Ks and Es intriguingly absent from SEQ ID NOS: 1 & 7 (S1). The theme of charged residues continues with the EPL001 Extension (SEQ ID NO: 5), where the only EPL001 residue lacking is E, with a blank x being returned by the sequencing instead.

Among the assumptions here are that the Edman reagent behaved faithfully in attaching itself to α-amines; the main sequencing machine (Applied Biosystems Procise) was operating and was operated properly; and that its data were interpreted correctly to yield the EPL001 sequence once in full (SEQ ID NO: 3) and six other times in part (S1 Table 1). The recurrence of residues (e.g. M, P, V & F) in full or near alignment argues for a degree of reliability within a picture of chemical sequencing travails, while the in-full EPL001 sequence data record (from The Babraham Institute, Cambridge, UK) was validated in a second independent assessment (personal communication, Will Mawby, University of Bristol, Bristol, UK).

Sequential release of N-terminal amino acids as their phenylthiohydantoin (PTH) derivatives is the basis of automated Edman degradation (Edman, 1950; Smith, 2001). The Edman reagent is phenyl isothiocyanate. Under mildly alkaline conditions this reacts with the N-terminal amine of a protein (anchored to a solid phase). The sulphur in the resulting derivative attacks the carbonyl carbon of the N-terminal amino acid to form a cyclical phenylthiocarbamoyl derivative, which is cleaved off as a thiazolinone compound after breaking of the peptide bond on the introduction of a mild acid (TFA). This is extracted into organic solvent and treated further with acid to form the more stable PTH-amino acid derivative, which is identified using liquid chromatography. The process is then repeated to identify the second amino acid and so on. The protein-immobilizing membrane used throughout the present studies was polyvinylidene difluoride (PVDF). Proteins bind to this via hydrophobic and dipole interactions. A strong interaction can be anticipated between PVDF and sSgII-70.

EPL001 numbered for sSgII-70 is M_1_K_7_P_8_L_2_T_4_G_5_K_3_V_9_K_11_E_6_F_10_∙N_68_N_69_I_70_. Notwithstanding the concept of Edman Nonsequentialism, out of order sequencing, the first residue of sSgII-70 is in fact read in Cycle 1 (S8 Fig. 1) accurately as methionine, as in EPL001’s **M**_1_K_2_P_3_L_4_T_5_G_6_K_7_V_8_K_9_E_10_F_11_N_12_N_13_I_14_ and sSgII-70’s **M**_1_L_2_K_3_T_4_G_5_E_6_K_7_P_8_V_9_F_10_K_11_. After M1 the second residue in the EPL001 readout is a lysine (Cycle 2). This is evidently sSgII-70’s K7 (see S2), the start of a spiral connotation (S8 Figs. 2 & 3). A true sequential reading should have next yielded sSgII-70’s L2. Why did a true sequential reading not occur? Two reasons can be adduced: the Edman reagent is subject to steric hindrance in regard to L2 and the acid used to release M1 simultaneously hydrolyses the peptide bond between the ionically conflicted E6 & K7, providing a free α amine group on K7. So, at the end of **Cycle 1** M1 has been read and two free α amines are simultaneously exposed: one each on L2 & K7. **Cycle 2** involves the reading of K7. This releases the N-terminal amine of P8, which residue is duly read in **Cycle 3**. In **Cycle 4** L2 is at last read, which discloses that it is P8 that has been providing the steric hindrance stopping the Edman reagent accessing L2. L2 is read in preference to V9, which after the taking of P8 has a free amine. So now we have EPL001’s **MKPL**TGKVKEFNNI. At the start of **Cycle 5** two residues have free amines: K3 (released by L2) & V9. Which is read? Neither. The machine instead chooses T4. This implies that the acid hydrolysis that released L2 to be read also at the same time broke the peptide bond between K3 and T4, anomalously, displaying the amine on the latter. The taking of T4 exposes G5 and this is taken next, **Cycle 6**, yielding **MKPLTG**KVKEFNNI. At the start of **Cycle 7** free α amines are present on the following remaining residues: K3, E6 (released by the taking of G5) and V9. K3 is taken next. Then at last V9 is taken to complete **Cycle 8**. The primary sequence deduction regarding sSgII-70 sees V9 as being peptide bonded to F10 (see paper, Discussion). So, the removal of V9 exposes the α amino group on F10. Yet F10 is not taken next, in **Cycle 9**, presumably because of steric considerations. Instead, K11 is taken as EPL001’s ninth residue, yielding **MKPLTGKVK**EFNNI. Next to be taken is E6, in **Cycle 10**, followed in **Cycle 11** by F10. After 11 cycles of Edman degradation this sequence has eventuated: **MKPLTGKVKEF**NNI. The remaining three residues, NNI, are hypothesized to be C-terminal in sSgII-70. They are physically contiguous to sSgII-11, by virtue of conjectured cross-linking, rather than a sequential continuation of sSgII-11. The acid hydrolysis that releases F10 must have broken the peptide bond N-terminal of N68, allowing this to be read in **Cycle 12**, then N69 and I70, in **Cycles 13 & 14** respectively. The EPL001 sequence is now complete: **MKPLTGKVKEFNNI**.

In the S8 Fig. 1 scheme just outlined there are four out-of-sequence peptide bond cleavages, all having a lysine association (with the anomalously available amine shaded): E =K (Cycle 1), =K =T (Cycle 4), =F =K (Cycle 8) and the non-contiguous (K) •=N (Cycle 9). A transformation can be achieved into EPL001 from sSgII-14 by moving the four charged residues, three Ks and E, together with P (S8 Fig. 4). The probability of choosing one uncharged residue of any kind from sSgII-14 and the four charged residues is about 1 in 200. The probability of choosing proline specifically as the uncharged residue, together with the four charged residues is about 1 in 2000 (S2). To these observations of ‘anomalous lysines’ can be added others. The Beale 4, from maximally purified ovine material, is P∙V∙FN (SEQ ID NO: 4 of S1 Table 1). The first three of these residues are sequential in the proposed sSgII-70 input sequence. In the EPL001 output sequence in contrast these are ranged at intervals as xx**P**xxxx**V**xx**F**Nxx. A spiral structure is connoted by this gapping. P, V & F are clustered on the righthand side of the double spiral stylisation of S8 Fig. 3, with N hypothesized to be nearby, non-contiguously. The Beale 4 reading yielded L & K as ‘under signals’ to the EPL001-matching V & F: xxPxxxx**V/L**xx**F/K**Nxx. The ‘under pair’ of L & K and the ‘over pair’ of V & F in the Beale 4 sequence occupy adjacent positions in sSgII-11: M**LK_3_**TGEK_7_P**VF**K_11_. This takes the K in the Beale 4 as sSgII-11’s K3, supporting the target sequence as LK, but the lysine could be sSgII-11’s K11, supporting the endogenous sequence as FK. In the S8 Fig. 1 scheme of ‘EPL001 from sSgII-14’, V & L are deduced to be available together for the Edman reagent and separately F & **K11** are deduced to display co-availability. Whether the Beale 4’s ‘under K’ is sSgII-14’s K3 or K11, the residues V, L, F & K_3/11_ are clustered together on the right of the S8 Fig. 3 double spiral stylisation. So, the Beale 4’s V & F are in register with the V & F in EPL001 and each of these two Beale 4 residues is paired with co-available residues, L & K, these having free α amines in a manner compatible with the S8 Fig. 1 scheme. The likelihood of this dual positional pairing arising by chance is 1 in 167 (S2).

Another duplicate reading involves lysine. When the Second Sighting sequence was obtained (SEQ ID NO: 2, S1) there was a mixed signal at position 2: M**L/K**P. After the taking of M1 in Cycle 1, there is not only the expected free α amine on the next residue, L2, but also, it has been surmised, on K7, which is taken in preference to L2 in Cycle 2. The hybrid N-terminal signal of the Second Sighting invokes sSgII-14’s **MLK** and EPL001’s **MKP**.

Chemical sequencing can occasionally give an incorrect reading, but nothing on the scale observed here in the form of Edman Nonsequentialism. Might post-translational modification (PTM) be important in this context? The MS data provide no evidence for additive PTMs (author JEH data), which can indeed disrupt Edman degradation, as they can tryptic digests, western blotting and ELISAs. Instead, the picture here is of faithful Edman degradation compromised by aberrant peptide bond cleavages, representing stepwise depolymerisation particularly associated with anomalous lysine availabilities, together with steric hindrance, due to sSgII-70 being a spiralised, probably cross-linked molecule (with a looped back polyanionic tail) contorted onto the reaction membrane.

**References**

Edman P (1950) Method for determination of the amino acid sequence in peptides. *Acta Chemica Scandinavica,* **4**, 283-293

Smith, JB (2001) Peptide sequencing by Edman degradation. *Encyclopedia of Life Sciences*, Wiley Online Library; DOI: 10.1038/npg.els.0002688
